# Association of interleukin 2, interleukin 12, and interferon-γ with intervertebral disc degeneration in Iranian population

**DOI:** 10.1186/s12881-020-01081-3

**Published:** 2020-07-03

**Authors:** Sara Hanaei, Sina Abdollahzade, Maryam Sadr, Mohammad Hossein Mirbolouk, Ehsan Fattahi, Alireza Khoshnevisan, Nima Rezaei

**Affiliations:** 1 Molecular Immunology Research Center, Tehran University of Medical Sciences, Tehran, Iran; 2 Research Center for Immunodeficiencies, Children’s Medical Center, Tehran University of Medical Sciences, Dr Qarib St, Keshavarz Blvd, Tehran, 14194 Iran; 3 Network of Immunity in Infection, Malignancy and Autoimmunity (NIIMA), Universal Scientific Education and Research Network (USERN), Tehran, Iran; 4 Division of neurosurgery, Department of surgery, Rajayi Hospital, Qazvin University of Medical Sciences, Qazvin, Iran; 5 Department of Neurosurgery, Shariati Hospital, Tehran University of Medical Sciences, Tehran, Iran; 6 Department of Immunology, School of Medicine, Tehran University of Medical Sciences, Tehran, Iran

**Keywords:** Intervertebral disc degeneration, Single nucleotide polymorphism, Interleukin 2, Interleukin 12, Interferon γ, Immunogenetics, Cytokine

## Abstract

**Background:**

Intervertebral disc degeneration (IVDD) is an age-related degenerative disease, presenting with low back pain or radicular pain. The inflammatory changes would occur in discs in the process of IVDD. Therefore, the inflammatory and anti-inflammatory cytokines, as well as their respective genes, have been proposed to play roles in pathophysiology of disease. This study has been conducted to elucidate the role of IL-2, IL-12, and IFN-γ single nucleotide polymorphisms (SNP) in this disease.

**Method:**

Seventy-six patients who were diagnosed with IVDD and 140 healthy controls who complied with eligibility criteria were included. A total volume of 5 cc peripheral blood was obtained from each participant to investigate the IL-2 + 166G/T, IL-2 -330G/T, IL-12 − 1188A/C, and IFN-γ +847A/T SNPs through PCR-SSP method.

**Results:**

The ‘TG’ and ‘TT’ genotypes of IL-2 − 330G/T polymorphism were significantly more common among patients and healthy controls respectively. The ‘GT’ and ‘TT’ haplotypes of IL-2 (comprised of -330G/T, and + 166G/T SNPs) were also more common among patients and controls respectively.

**Conclusion:**

This study indicated the significant role of IL-2 genotypes and haplotypes in IVDD. These SNPs were differently distributed in patients and controls. Therefore, alteration in the structure of IL-2 gene could play an important role in pathophysiology of IVDD.

## Background

Intervertebral disc degeneration (IVDD) is one of the common causes of discogenic low back pain, which is considered a frequent health problem in adults. This disease affects the patients’ quality of life and also has the negative impact on the length of their productive life [1]. Although the mechanical load on the discs and aging are considered as the most important causes of IVDD, genetic and immunologic predispositions have been widely discussed especially in the past 2 decades [1]. Accordingly, the individuals with specific genetic predispositions, such as single nucleotide polymorphisms (SNP) of immunologic modulators and cytokines, would be more prone to develop IVDD. Also they might be affected with severe grades of IVDD or develop disease in younger ages [1].

Generally, innate immunity and inflammation play important roles in IVDD occurrence. The inflammation would cause destructive damages to the discs through its mediators including pro-inflammatory cytokines and also other cytokines (interleukins, interferons), enzymes and growth factors. Interleukin 2 (IL-2), located on 4q27, is mainly produced by mature T-cells and participates in development of T-cells and B-cells, as it can function as a growth factor for them [2].

The gamma interferon (IFN-γ), which is located at 12q15, is a cytokine with important roles in immunity and immune related diseases. Its expression would remarkably increase during the inflammatory reactions [2]. In the process of disc degeneration, and disc herniation, the IFN-γ is one of the inflammatory components which is upregulated in disc nucleous pulposus (NP), and affects tissue-specific macrophages in NP [3]. When produced by T helper 1 lymphocytes in the discs, IFN-γ participates in macrophage activation, which could be considered as an immune response to herniation of NP [4]. Moreover, IFN-γ plays a role in pathogenesis of neuropathic pain as well [5]. As the IFN-γ is upregulated in neuroinflammation, as well as affecting the nociceptive neurons, any structural change in its gene, which results in higher expression, could possibly play a role in pathogenesis of disease. Therefore, some specific SNPs of this gene have been found to affect its expression levels [3].

Interleukin 12 (IL-12) is a cytokine with main role of connecting innate and acquired immunity [2]. Together with other factors and immune-related molecules, this cytokine plays a role in IVDD as well. Similar to IFN-γ, the IL-12 level was found higher in disc fragments caused by disc herniation [4].

As the important role of genetic and immunologic predisposition to IVDD, we have investigated the association of IVDD with different cytokines so far, including the proinflammatory cytokines, interleukin 4, 10, and TGF-β [6–8]. Although the expression levels of some other cytokines including IL-2 and IFN-γ have been of interest in some other studies, the association of their SNPs have not been investigated yet. Therefore, the aim of current study was to evaluate the association of IL-2, IL-12, and IFN-γ SNPs in Iranian patients with IVDD, as well as their association with post-operative pain reduction in this population.

## Method

### Patient selection

Seventy-six adult patients of both sexes with history of chronic low back pain who were diagnosed with intervertebral disc degeneration, and indicated for surgical intervention have been included in this study. The diagnosis was confirmed with both clinical manifestations and lumbosacral MRI. Patients with other etiologies of low back pain including trauma, malignancies, scoliosis, infections, spondylolysthesis, spondylolysis, and inflammatory disease affecting spine were detected ineligible to participate in this study. The control group (*N* = 140) has been selected among adult subjects of both sexes and same ethnicity without history of chronic low back pain or intervertebral disc degeneration. Participation in the study has been voluntary for all study subjects. Written informed consent to participate was obtained from all study participants before recruitment and sampling. The study has been approved by Ethics Committee of Tehran University of Medical Sciences.

### Assessment of visual analogue scale (VAS) and Oswestry disability index (ODI)

The VAS and ODI were assessed in patients, at the time points of pre-operative, 2 months post-operative and 6 months post-operative. In order to assess the VAS, the patients were requested to score their pain on scale of 1–10. Score 1 indicated the lowest degree of pain and score 10 indicated the highest degree. The ODI was assessed using a questionnaire as described before [9]. Then the difference of measurements between two time points were analyzed in order to investigate if a specific gene variant was associated with significant change in VAS or ODI.

### Blood sampling, DNA extraction, and polymerase chain reactions (PCR)

A total volume of 5 cc peripheral blood was obtained from all patients and controls in falcon tubes containing EDTA, and stored in − 20^°c^ prior to DNA extraction. Previously described by Loparev V. et al. [10], genomic DNA was extracted with Phenol-Chloroform method. In order to detect the quality of extracted DNA, the optical density (OD) and 260/280 ratio were measured with NanoDrop. Four SNPs including IL-2 + 166G/T, IL-2 -330G/T, IL-12 − 1188A/C, and IFN-γ +847A/T have been genotyped through Sequence Specific Prime PCR (PCR-SSP) method using Cytokine CTS-PCR-SSP TRAY KIT (University Clinic Heidelberg, Heidelberg, Germany) protocol. In brief, the 96-well reaction plate of cytokine kit contained a total volume of 10 μl/well solution, which was comprised of 1.44 μl Master Mix, 3.43 μl dH_2_O, 0.57 μl DNA, and 0.04 μl Taq enzyme. The PCR reactions have been performed accordingly to the kit protocol.

### Statistical analysis

Distributions of qualitative variables, such as alleles and genotypes, were reported as frequencies. The association between two qualitative variables was assessed through calculating odds ratio (OR) and Chi^2^ test. The respective *P*-value was determined as indication of significance and *P* < 0.05 was considered statistically significant in all tests.

## Results

### Association of cytokine (IL-2, Il-12, and IFN-γ) allele and genotype distributions with intervertebral disc degeneration

Genotype distribution of IL-2 promoter polymorphism (− 330 G/T) was significantly associated with IVDD, as ‘TG’ genotype was 2.05 times more common among patients (*P* = 0.05), while the ‘TT’ genotype was more frequent among healthy subjects (*P* = 0.03). However, other investigated polymorphisms failed to show any significant association with IVDD in either allele or genotype distribution (Table 1).

**Table 1:** The frequencies of cytokine alleles and genotypes in IVDD patients and healthy controls

Cytokine	Position	Alleles/ Genotypes	IVDD N (%)	Control (***N*** = 140) N (%)	***P***-Value	OR	95% CI
IL-12 (*N* = 71)	-1188 (rs3212227)	A	103 (72.5)	204(72.9)	1.00	0.98	0.63–1.55
		C	39 (27.5)	76(27.1)	1.00	1.02	0.65–1.58
		AA	40 (56.3)	72(51.4)	0.56	1.22	0.69–2.16
		CA	23 (32.4)	60(42.9)	0.18	0.64	0.35–1.16
		CC	8 (11.3)	8(5.7)	0.17	2.09	0.75–5.84
IFN-γ (*N* = 74)	+ 874 (rs2430561)	A	85 (57.4)	140(50.7)	0.22	1.31	088–1.96
		T	63 (42.6)	136(49.3)	0.22	0.88	0.51–1.31
		AA	26 (35.1)	43(31.2)	0.65	1.19	0.66–2.18
		AT	33 (44.6)	54(39.1)	0.47	1.25	0.71–2.19
		TT	15 (20.3)	41(29.7)	0.15	0.60	0.31–1.18
IL-2 (*N* = 74)	-330 (rs2069762)	G	68 (46.0)	110(39.6)	0.21	1.31	0.88–1.97
		T	80 (54.0)	168(60.4)	0.21	0.76	0.50–1.13
		GG	4 (5.4)	8(5.8)	1.00	0.94	0.27–3.22
		**TG**	**60 (81.1)**	**94(67.6)**	**0.05**	**2.05**	**1.03–4.06**
		**TT**	**10 (13.5)**	**37(26.6)**	**0.03**	**0.43**	**0.20–0.93**
IL-2 (*N* = 74)	+ 166 (rs2069763)	G	113 (76.4)	219(78.8)	0.62	0.87	0.54–1.40
		T	35 (23.6)	59(21.2)	0.62	1.15	0.71–1.85
		GG	40 (54.1)	82(59)	0.56	0.82	0.46–1.44
		GT	33 (44.6)	55(39.6)	0.56	1.23	0.69–2.17
		TT	1 (1.4)	2(1.4)	1.00	0.94	0.08–10.52

### Association between cytokine haplotype distribution and intervertebral disc degeneration

Considering two polymorphisms of IL-2 together (the -330G/T and + 166G/T SNPs), two haplotypes were differently distributed in patients and controls. While ‘GT’ haplotype was more frequent among patients, the ‘TT’ haplotype was more common among healthy subjects (OR = 0.44, *P* = 0.009) (Table 2).

**Table 2:** The frequencies of IL-2 haplotypes in IVDD patients and healthy controls

Cytokine	Haplotype*	IVDD N (%)	Control (***N*** = 140) N (%)	***P***-Value	OR	95% CI
IL-2	GG	48 (32.2)	107(38.8)	0.20	0.75	0.49–1.14
	TG	65 (43.6)	112(40.6)	0.60	1.13	0.76–1.69
	TT	**15 (10.1)**	56(20.3)	**0.009**	**0.44**	**0.24–0.81**
	GT	**20 (13.4)**	1(0.3)	**–**	**–**	**–**

*Reference: The IL2 haplotype was comprised of -330 T/G SNP (rs2069762), and + 166G/T SNP (rs2069763)

### Association of Il-2 and IFN-γ polymorphisms with Oswestry disability index and visual analogue scales

The allele and genotypes of IL-2 and IFN-γ were considered for evaluating their association with post-operative pain reduction. The association between each allele or genotype and pain indices are shown in detail in Tables 3, 4, 5 and 6. Among them, only IL-2 -1188A/C indicated significant association with 6-month post-operative ODI reduction.

**Table 3:** Association between cytokine genotypes and 2 months or 6 months postop OSW changes in IVDD patients

Cytokine	Position	Genotype	2 months postop		6 months postop	
			MD ± SD (MD)	***P***-value	MD ± SD (MD)	***P***-value
IL-12	-1188	AA	−18.75 ± 5.69	0.19	−24.54 ± 5.60	0.07
		CA	−21.36 ± 6.01		−27.59 ± 3.69	
		CC	−21.50 ± 5.08		−26.50 ± 5.16	
IFN-γ	+ 874	AA	−20.08 ± 5.44	0.99	−25.82 ± 4.54	0.98
		AT	−20.11 ± 5.92		− 25.92 ± 5.18	
		TT	−20.35 ± 6.12		− 25.57 ± 6.33	
IL-2	−330	GG	−27.50 ± 0.70	0.12	−28.00 ± 2.82	0.82
		TG	−19.54 ± 5.90		−25.80 ± 5.27	
		TT	−21.37 ± 4.03		−25.50 ± 4.75	
IL-2	+ 166	GG	−20.66 ± 5.38	0.33	−26.21 ± 4.27	0.27
		GT	−19.12 ± 6.14		−25.19 ± 5.85	
		TT	−26.00		−33.00

**Table 4:** Association between cytokine genotypes and 2 months or 6 months postop VAS changes in IVDD patients

Cytokine	Position	Genotype	2 months postop		6 months postop	
			MD ± SD (MD)	***P***-value	MD ± SD (MD)	***P***-value
IL-12	−1188	AA	−4.37 ± 1.42	0.52	−5.62 ± 1.29	0.49
		CA	−4.77 ± 1.10		− 5.90 ± 1.06	
		CC	−4.50 ± 0.83		−6.16 ± 1.47	
IFN-γ	+ 874	AA	−4.65 ± 1.19	0.49	−5.86 ± 1.09	0.92
		AT	−4.37 ± 1.49		−5.74 ± 1.48	
		TT	−4.85 ± 0.94		−5.85 ± 0.94	
IL-2	−330	GG	−6.00 ± 0	0.22	− 6.00 ± 0	0.88
		TG	−4.56 ± 1.31		−5.83 ± 1.21	
		TT	−4.25 ± 0.88		−5.62 ± 1.50	
IL-2	+ 166	GG	−4.57 ± 1.27	0.90	−5.84 ± 1.12	0.18
		GT	−4.58 ± 1.31		−5.70 ± 1.29	
		TT	−4.00		−8.00

**Table 5:** Association between cytokine allele frequencies and 2 months or 6 months postop OSW changes in IVDD patients

Cytokine	Position	Allele	2 months postop		6 months postop	
			MD ± SD (MD)	***P***-Value	MD ± SD (MD)	***P***-Value
IL-12	−1188	A	−19.35 ± 5.81	0.07	−25.23 ± 5.33	**0.05**
		C	−21.41 ± 5.55		−27.20 ± 4.13	
IFN-γ	+ 874	A	−20.09 ± 5.54	0.89	− 25.86 ± 4.72	0.89
		T	−20.23 ± 5.91		−25.74 ± 5.68	
IL-2	−330	G	−20.08 ± 6.04	0.90	−25.94 ± 5.14	0.81
		T	−19.95 ± 5.54		−25.73 ± 5.09	
IL-2	+ 166	G	−20.17 ± 5.62	0.58	−25.88 ± 4.80	0.83
		T	−19.54 ± 6.17		−25.66 ± 5.97

**Table 6:** Association between cytokine allele frequencies and 2 months or 6 months postop VAS changes in IVDD patients

Cytokine	Position	Allele	2 months postop		6 months postop	
			MD ± SD (MD)	***P***-Value	MD ± SD (MD)	***P***-Value
IL-12	−1188	A	−4.46 ± 1.35	0.41	−5.68 ± 1.24	0.20
		C	−4.67 ± 1.00		−6.00 ± 1.18	
IFN-γ	+ 874	A	−4.54 ± 1.30	0.75	−5.82 ± 1.23	0.92
		T	−4.61 ± 1.25		−5.80 ± 1.22	
IL-2	−330	G	−4.66 ± 1.32	0.45	−5.84 ± 1.17	0.78
		T	−4.49 ± 1.22		−5.78 ± 1.26	
IL-2	+ 166	G	−4.57 ± 1.27	0.90	−5.80 ± 1.16	0.85
		T	−4.54 ± 1.27		−5.84 ± 1.37	

## Discussion

To our knowledge, the current study was one of the firsts to investigate the association of cytokine polymorphisms including IL-2 (− 330 T/G, + 166G/T), IL-12 (−1188A/C) and IFN-γ (=874A/T) with intervertebral disc degeneration (IVDD) in Iranian patients. Among all the investigated SNPs, only the IL-2 -330 T/G was significantly associated with disease, and was twice more common in patients than controls. Besides, the haplotype of IL-2 (− 330, + 166) was also significantly associated with IVDD, as the ‘GT’ haplotype was more frequent in IVDD patients. However, other investigated SNPs failed to show any association with IVDD in this population.

The role of immunity, as well as the cytokines of interest in this study, were investigated in a number of sturdies so far. Although the biomarker levels of these cytokines were investigated in a number of human studies or animal models, the role of SNPs were not widely investigated in the literature.

The IFN-γ level would significantly change in low back pain. A recent investigation in 2019, indicated higher levels of IFN-γ in acute low back pain compared with chronic low back pain or healthy subjects. Interestingly, the IFN-γ level was not significantly different between chronic low back pain and asymptomatic individuals [11]. A considerable number of patients with IVDD who experience chronic low back pain may require surgical intervention, nevertheless, some of them may not response to this treatment either. The IFN-γ expression level was associated with increased numeric rating scale. Accordingly, higher levels of IFN-γ were detected in patients who did not respond to surgery. Spinal cord stimulation might be considered as an option for improving the pain and quality of life in these patients. However, in an investigation, IFN-γ level was not significantly altered after the stimulation [5]. IFN-γ levels were associated with acute low back pain, although its association with chronic low back pain was not significant [11]. While the IFN-γ SNP did not show significant association with postoperative pain reduction in our study, the ‘A’ allele of IFN-γ rs2069705 was remarkably associated with higher ODI score in a study, as the patients with ‘AA’ and ‘GA’ genotypes had significantly higher ODI scores. In addition, the ‘AG’ and ‘GG’ genotypes of IFN-γ rs2069718 were associated with higher ODI scores in this Norwegian population [3].

The IL-12 mainly functions together with other cytokines such as IFN-γ, and the levels of these cytokines were different in fragments of herniated discs and degenerative disc tissue. Accordingly, both IL-12 and IFN-γ, as well as other cytokines (IL-4, IL-6) indicated higher levels in the fragments of herniated discs [4]. On the other hands, the expression levels of these cytokines were not remarkably different between nucleous pulposus and anulus fibrosus of healthy discs obtained from autopsies [4].

Although the IL-2 plays an important role in inflammatory processes, its expression level was not significantly different between acute or chronic low back pain, or even the asymptomatic subjects [11]. Moreover, the IL-2 level was not associated with VAS scores either in acute or chronic low back pain [11]. In a study measuring the levels of different cytokines in low back pain, IL-2 levels in the sera of patients with low back pain were remarkably lower than controls, similar to other factors including IL-6, IL-4, and MMP-1. On the other hands, the serum levels of IL-12 and IFN-γ were not significantly different between patients and healthy controls [12]. However, another study indicated higher levels of IL-2 and IL-12 in degenerated disc tissue compared to controls [13]. Another study confirmed this finding, as both the mRNA level and protein level of IL-2 were higher in prolapsed NP cells than controls [14].

In an animal model of disc herniation in rats, presence of NP and IFN-γ in the dorsal nerve root of animals resulted in remarkable higher activity of nociceptive neurons [3]. The intervertebral disc degeneration would affect different components of spinal cord, in addition to disc itself. The inflammatory process and destructive damages would also affect the spinal components such as epidural space. It could be explained as the inflammation in this component would have compressive effect on nerve roots and cause more severe clinical manifestations. Accordingly, in a canine model of disc degeneration, the cytokine levels were assessed in different stages of disc extrusion. Importantly, the expression level of IL-2 was significantly decreased in disc extrusion, especially in the subacute stage [15]. However, another animal model in rabbits showed contradictory results, as the IL-2 level was significantly increased not only in degenerated disc tissue, but also in the sera of animals. Moreover, in that model, the effect of plasma vaporization ablation was evaluated for treatment of IVDD. The expression levels of IL-12, as well as some other cytokines such as IL-4 and TNF-α, were decreased after applying this method, which could indicate its effectiveness in treating IVDD [16]. The mRNA expression of IL-2 was also remarkably higher in another model of disc herniation in rats [17]. In an animal model of disc herniation in rats, dorsal root ganglions were exposed to NP, and epidural lavage indicated higher expression of IFN-γ, as well as other cytokines such as IL-4, IL-6, and TNF-α [18].

Taking limitations of current study to account, small sample size could be considered as one of them, and therefore, a larger sample size could improve random error or increase the study power. Meanwhile, the genetic factors may have co-effect on each other, and therefore a wider panel measuring the role of different cytokines and other genetic factors could be helpful if better understanding of the role of each factor. Moreover, it shall be considered that assessment of gene SNPs together with expression levels or protein levels would more confidently demonstrate the association between genetic factors and disease. As it was not applicable to measure the IL-2 and other cytokine levels in this study, it is recommended to be considered in the future studies. It should be also considered that the status of some SNPs could not be interpreted though electrophoresis gels in some samples, and therefore, those patients were not included in the analysis for that particular SNP.

## Conclusion

Cytokines variants including IL-2 -330 T/G could be considered as a predisposing factor for IVDD, as the ‘TG’ genotype was twice more common in Iranian adults with IVDD. As the importance of IVDD in daily lives of working population, understanding genetic predispositions could be invaluable to detect high risk individuals, and therefore, consider some preventive conditions for them, such as avoiding them to choose jobs which involve high load on lumbar discs. In addition, detecting the genetic factors which influence the response to treatment, could be helpful in approaching the patients. In other words, patients with a specific gene variant (exe. IL-12 -1188A/C) might benefit more from surgery, as their post-operative ODI were lower than pre-operative measurements.

## Data Availability

The data that support the findings of this study are available from the corresponding author (Nima Rezaei) but restrictions apply to the availability of these data, which were used under license for the current study, and so are not publicly available. Data are however available from the authors upon reasonable request and with permission of the corresponding author (Nima Rezaei). Meanwhile, it shall be noted that no dataset was generated or used for this project.

## Abbreviations

IFN-γ: Interferon gamma
IL-12: Interleukin 12
IL-2: Interleukin 2
IVDD: Intervertebral Disc Degeneration
NP: Nucleous Pulposus
OD: Optical Density
ODI: Owestry Disability Index
OR: Odds Ratio
PCR: Polymerase Chain Reactions
PCR-SSP: Sequence Specific Prime PCR
SNP: Single Nucleotide Polymorphisms
TNF-α: Tumor Necrosis Factor alpha
VAS: Visual Analogue Scale
